# Determining optimal mobile neurofeedback methods for motor neurorehabilitation in children and adults with non-progressive neurological disorders: a scoping review

**DOI:** 10.1186/s12984-022-01081-9

**Published:** 2022-09-28

**Authors:** Ahad Behboodi, Walker A. Lee, Victoria S. Hinchberger, Diane L. Damiano

**Affiliations:** Rehabilitation Medicine Department, National Institutes of Health, Bethesda, MD USA

**Keywords:** Brain computer interface, BCI, Rehabilitation, Stroke, Motor training, Brain state-dependent stimulation, Cerebral palsy, Neuroplasticity

## Abstract

**Background:**

Brain–computer interfaces (BCI), initially designed to bypass the peripheral motor system to externally control movement using brain signals, are additionally being utilized for motor rehabilitation in stroke and other neurological disorders. Also called neurofeedback training, multiple approaches have been developed to link motor-related cortical signals to assistive robotic or electrical stimulation devices during active motor training with variable, but mostly positive, functional outcomes reported. Our specific research question for this scoping review was: for persons with non-progressive neurological injuries who have the potential to improve voluntary motor control, which mobile BCI-based neurofeedback methods demonstrate or are associated with improved motor outcomes for Neurorehabilitation applications?

**Methods:**

We searched PubMed, Web of Science, and Scopus databases with all steps from study selection to data extraction performed independently by at least 2 individuals. Search terms included: brain machine or computer interfaces, neurofeedback and motor; however, only studies requiring a motor attempt, versus motor imagery, were retained. Data extraction included participant characteristics, study design details and motor outcomes.

**Results:**

From 5109 papers, 139 full texts were reviewed with 23 unique studies identified. All utilized EEG and, except for one, were on the stroke population. The most commonly reported functional outcomes were the Fugl-Meyer Assessment (FMA; n = 13) and the Action Research Arm Test (ARAT; n = 6) which were then utilized to assess effectiveness, evaluate design features, and correlate with training doses. Statistically and functionally significant pre-to post training changes were seen in FMA, but not ARAT. Results did not differ between robotic and electrical stimulation feedback paradigms. Notably, FMA outcomes were positively correlated with training dose.

**Conclusion:**

This review on BCI-based neurofeedback training confirms previous findings of effectiveness in improving motor outcomes with some evidence of enhanced neuroplasticity in adults with stroke. Associative learning paradigms have emerged more recently which may be particularly feasible and effective methods for Neurorehabilitation. More clinical trials in pediatric and adult neurorehabilitation to refine methods and doses and to compare to other evidence-based training strategies are warranted.

## Introduction

Neurofeedback training is a promising neurorehabilitation strategy for improving motor function that has emerged from the Brain Computer Interface (BCI) field. In contrast to bypassing voluntary motor control by linking the brain to a wearable device that provides movement, BCI-mediated neurofeedback training (BCI-NFT) in a rehabilitation context aims to harness and link brain activity during real or imagined movement to strengthen adaptive neural connections and thereby enhance motor capabilities. BCI applications were initially developed to enable individuals who are virtually unable to communicate or perform motor functional activities, e.g. those with locked in syndrome or Amyotrophic Lateral Sclerosis, to generate reliable brain activation signals representing their intent in order to control assistive technology. While the possibilities of exploiting brain control of devices are remarkable, the current reality is that these systems are mainly capable of producing a reliable signal to control an external device such as a wearable exoskeleton (e.g. to take a step) [1] or to discriminate among a limited group of preset options (e.g. to move the right or left hand) [2], and often require extensive training for proficiency that not all who attempt this can achieve [3]. Advances in collection, processing and identification or classification of brain signals associated with motor intent gleaned from these assistive or substitutive BCI technologies have led to the design of rehabilitative or restorative BCIs to provide a means of motor training for those with some degree of, or potential for, voluntary motor ability. BCI-NFT may be a particularly effective approach for individuals with neurological disorders who have limited to no residual motor activity [4, 5] and may not yet be able to benefit from existing effective rehabilitation strategies that require a baseline amount of active movement [5]. These patients, however, can still imagine or attempt to move and the associated brain activity can be used as the control command for BCI-NFT systems.

While BCI-NFT paradigms have been an active area of research in neurorehabilitation for approximately the last 15 years, neurofeedback has been utilized for decades for many clinical applications such as the treatment of attention deficit hyperactivity disorder [6], anxiety [7], depression [8], schizophrenia [9], autism spectrum disorder [10], drug addiction [11], insomnia [12], seizures [13], and pain management [14], among others. Neurofeedback is defined broadly as “a kind of biofeedback, which teaches self-control of brain functions to subjects by measuring brain waves and providing a feedback signal” [15]. Generally, when treating different behavioral conditions, specific brain signals are identified as targets for modulation in a specified direction, e.g. up or down regulation, with the intended training-induced change in brain activity presumed to be linked directly to a positive change in the target behavior. Since most individuals have little if any inherent awareness of their success in modulating brain activity, neurofeedback training involves pairing their performance with an external, often visual, cue that gives them feedback on their success [16]. Based on the principle of operant conditioning, individuals gradually learn to reinforce the modulations that were deemed most successful and eliminate those that were less or unsuccessful. While some studies demonstrate that neurofeedback can be effective in certain disorders, the preponderance of evidence is inconsistent and neurofeedback for most applications is still largely considered an alternative treatment [15].

In neurorehabilitation, external neuromodulation using transcranial magnetic stimulation (TMS) or transcranial direct current stimulation (tDCS) paired with motor training has demonstrated effectiveness in stroke and cerebral palsy (CP) [17, 18]. Also a form of neuromodulation, BCI-NFT instead requires the user to activate their own neural pathways. This can be done using either a non-specific or specific approach. An example of a non-specific approach would be to identify any strategy that successfully modulates the desired rhythm and then use that conditioned response to control a cursor on a computer or a device, which is the classic approach to neurofeedback training. Although this type of neurofeedback, may activate central nervous system pathways, the induced plasticity is usually widespread and not specific to the target circuit and may also require much longer training [19, 20]. A second more task-specific approach would be to think about or perform a target movement or movements which then couples movement-related brain-states to time-correlated sensory feedback; i.e. a component of the brain signal produced by the motor intention or attempt is extracted in real-time and used to activate an assistive device. This paradigm, also referred to as associative learning, aims to both augment the voluntary motor response and reinforce the link between motor function and the brain if the sensory feedback is appropriately timed to arrive during the most active state of brain activation. Furthermore, this type of training has the potential to increase the intensity or efficiency of rehabilitation by providing high quality repetitive motion and augmented feedback [21]. Therefore, BCI-NFT systems are being increasingly deployed for motor training [22–27]. These are also reaching a level of technological maturity whereby they can provide faster and more reliable feedback for rehabilitation applications [28–32].

The brain state used for generating the BCI generated feedback for motor training is either motor imagery (MI), i.e. imagining the target motion without execution, or motor attempt (MA), which may or may not result in overt movement depending on user’s capability. MI continues to be a commonly utilized option for the control of BCI-NFT systems [24, 25, 27, 33–35]. However, with MI, patients may have to actively suppress the movement of the target limb while imagining the movement, and it requires learning and prolonged concentration which might be difficult for very young or cognitively challenged individuals. A recent study in children with CP [36] used age-specific metaphoric instructions to simplify the MI task for the participants, indicating that this is possible; however, it has also been shown that even some healthy adults may not be able to learn how to control BCI systems using MI [37–39]. It is clearly more natural to attempt the movement as well as more verifiable [40]. When the goal of the neurofeedback therapy is strengthening or reestablishing a lost motor function, controlling the BCI system by attempting to move the target limb, rather than using MI, may improve outcomes because MA maximizes the similarities between the brain-state used to control the BCI and the functional task. Therefore, the plasticity induced by the training might be more pronounced and more likely to persist beyond the therapy period [41]. A recent review [42] concluded that using MA for BCI-NFT may be more effective than using MI (p=0.07) based on a comparison of two MA studies and seven MI studies. Although the sensorimotor loop is disrupted in patients with lost or limited voluntary movements due to neurological disorders, some accessible brain pathways may still exist [43]. Thus, rather than learning an effective MI strategy, MA appears to be a better approach where possible for motor rehabilitation to restore more normal timing of motor preparation, execution, and resultant peripheral input from the muscle effectors [43, 44] and to potentially form a stronger or new sensorimotor loop [4, 22].

Several narrative reviews have been published in recent years which discuss clinical outcomes, underlying mechanisms, or technical advances and challenges across a broad range of BCI and/or neurofeedback applications, many of which also mention their use for motor rehabilitation [21, 30, 45–48]. Other reviews have focused specifically on the effects of BCI-NFT in motor rehabilitation; including four systematic reviews addressing the stroke population. The review by Carvalho et al. focused on upper limb recovery and only included randomized controlled trials (RCTs) that reported at least one clinical outcome (n = 9 studies) [49]. Similarly, Bai et al. performed a meta-analysis on the effects of BCI-NFT on the upper limbs from 33 studies including 18 single-group studies and 15 with a comparison group [42]. Baniqued et al. reviewed 30 studies on BCI-robots for hand rehabilitation, 19 of which were related to preclinical development of these systems and 11 of which were on their use in stroke [2]. Among these systematic reviews, the meta-analysis by Cervera et al. [50] is the only one that reviewed the effect of BCI-NFT on both upper and lower limbs in the stroke population. Studies which used BCI for both control and intervention groups were excluded from their review, resulting in nine RCTs included in this review. Both of the meta-analyses by Cervera et al. [50] and Bai et al. [42] focused on the Fugl-Meyer Assessment (FMA) score, showing positive trends in favor of the BCI-NFT group. Bai et al. showed a medium effect size favoring BCI-NFT for improving upper extremity function after intervention, while the long-term effects reported in five studies were not significant [42]. In Cervera et al. the standardized mean differences in the FMA scores were higher in neurofeedback versus control groups, although the between group differences did not reach the threshold of clinical significance [50].

Although not definitive, the effectiveness of BCI-NFT in stroke as reported in these meta-analyses generally appears promising. However, it is also notable that results across studies within these reviews varied considerably as did the methods, with no one method touted as superior. The aim of this review, therefore, is to evaluate which BCI-NFT methods appear to be associated with greater or poorer effectiveness in improving motor skills, to potentially identify the key components for successful interventions. Since our primary focus is on methodological differences and their associations with outcomes, rather than clinical effectiveness per se, we decided to perform a scoping review. In contrast to previous reviews, here we chose to only include those studies where the participants were instructed to attempt the target movement (MA), not simply to imagine it. We also limited our focus to those using non-invasive brain imaging techniques such as EEG and functional near-infrared spectroscopy (fNIRS) because these are far more accessible in clinical practice and more ecologically valid since everyday movements can be practiced in upright and more naturalistic settings. We aimed to include all studies on populations with neurological conditions that are non-progressive and therefore have the potential to respond to rehabilitation strategies aiming to improve motor capabilities (e.g. adults and children post-stroke or with cerebral palsy, among others). The ultimate goal of this review is to provide recommendations to the field of neurorehabilitation on the neurofeedback techniques and protocols most likely to improve the outcome of motor rehabilitation in those with non-degenerative neurological disorders for future implementation into therapy settings.

## Methods

This scoping review was registered in the Open Science Framework database (registration ID: DOI 10.17605/OSF.IO/2KHRX). and was conducted according to the Preferred Reporting Items for Systematic Reviews and Meta-Analyses extension for Scoping Reviews (PRISMA-ScR) Checklist [51]. Our research question was formulated for scoping reviews to include the intended Population, Concept, and Context (PCC) [52]: For persons with non-progressive neurological injuries who have the potential to improve their voluntary motor control (Population), which non-invasive mobile BCI-NFT methods, if any, demonstrate or are associated with improved motor outcomes (Concept) for Neurorehabilitation applications (Context)?

### Search strategy

A medical librarian at the National Institutes of Health was consulted to develop the optimal search strategy to address our research question. A title and abstract keyword search was conducted utilizing the following search terms and general strategy adapted as needed for the PubMed, Web of Science, and Scopus databases: “motor” AND “Brain Computer Interface” OR “BCI” OR Neurofeedback” OR “BMI” OR “EEG biofeedback”. Only articles published in the English language were considered. There was no restriction on the date of publication with April 15, 2021 as the final search date.

### Eligibility criteria

All clinical studies on the application of non-invasive, mobile (i.e. EEG or fNIRS) BCI-NFT for motor neurorehabilitation of individuals (children or adults) with non-progressive neurological injuries (e.g., stroke or CP) were included. We excluded studies that only enrolled healthy participants or those with progressive neurological conditions such as Parkinson’s Disease or Amyotrophic Lateral Sclerosis. Studies using Magnetic Resonance Imaging (MRI) or magnetoencephalography (MEG) to deliver neurofeedback were excluded. Systematic or scoping reviews were not included; however, reference lists of relevant reviews were scanned for studies that may not have been captured in the initial search. Further criteria for inclusion were that the interventions had to utilize a feature of the participants’ cortical activity within the training session, and that participants had to be attempting to perform a voluntary motor task. Studies in which participants were using motor imagery or action observation to generate the brain activation signals used for neurofeedback were excluded. Finally, since the goal was to examine how methodological differences might affect motor outcomes, only studies that reported these measures were included.

### Selection criteria and data charting

Duplicates were initially eliminated within ENDNOTE. Titles and abstracts were screened independently in EndNote by two authors (AB, DD) to remove additional duplicates and to identify studies that potentially met the inclusion criteria. Disagreements on which articles to retain were resolved through discussion. Full texts of all potentially eligible papers were independently assessed by the same two review authors, with disagreements again resolved through discussion. Reference lists of the final set of papers, as well as of relevant systematic reviews, were also scanned to ensure that no studies were missed. Then, three authors (AB, VH, WL) extracted data independently from the final group of studies satisfying all inclusion and exclusion criteria, with each assigned a group of articles to extract data from using a comprehensive pre-piloted data extraction form, in Google Sheets format, and a group to verify data extracted by another author.

### Data items

Extracted information included: study population and participant demographics; number of participants in intervention and control conditions, motor task performed during BCI-NFT and whether it was an upper limb or lower limb task, control condition, dosage of BCI-NFT, cortical activity feature(s) extracted to generate the feedback, cortical region(s) the feature was selected from, signal processing technique used for extracting the feature(s) and generating feedback, feedback timing, type of feedback (e.g., visual, robotic, functional electrical stimulation), whether any other additional training (e. g., conventional physical therapy) was provided before and/or after BCI-NFT , and finally, all reported motor outcome measures.

### Statistical analysis

Where possible, mean differences in motor outcome measures across specific feature categories (e.g. feedback types) were analyzed using a General Linear Model (GLM) or independent t-tests. Pearson correlation procedures were also used to relate specific training aspects or features to motor outcomes (p<0.05 for all analyses).

## Results

**Fig. 1:**
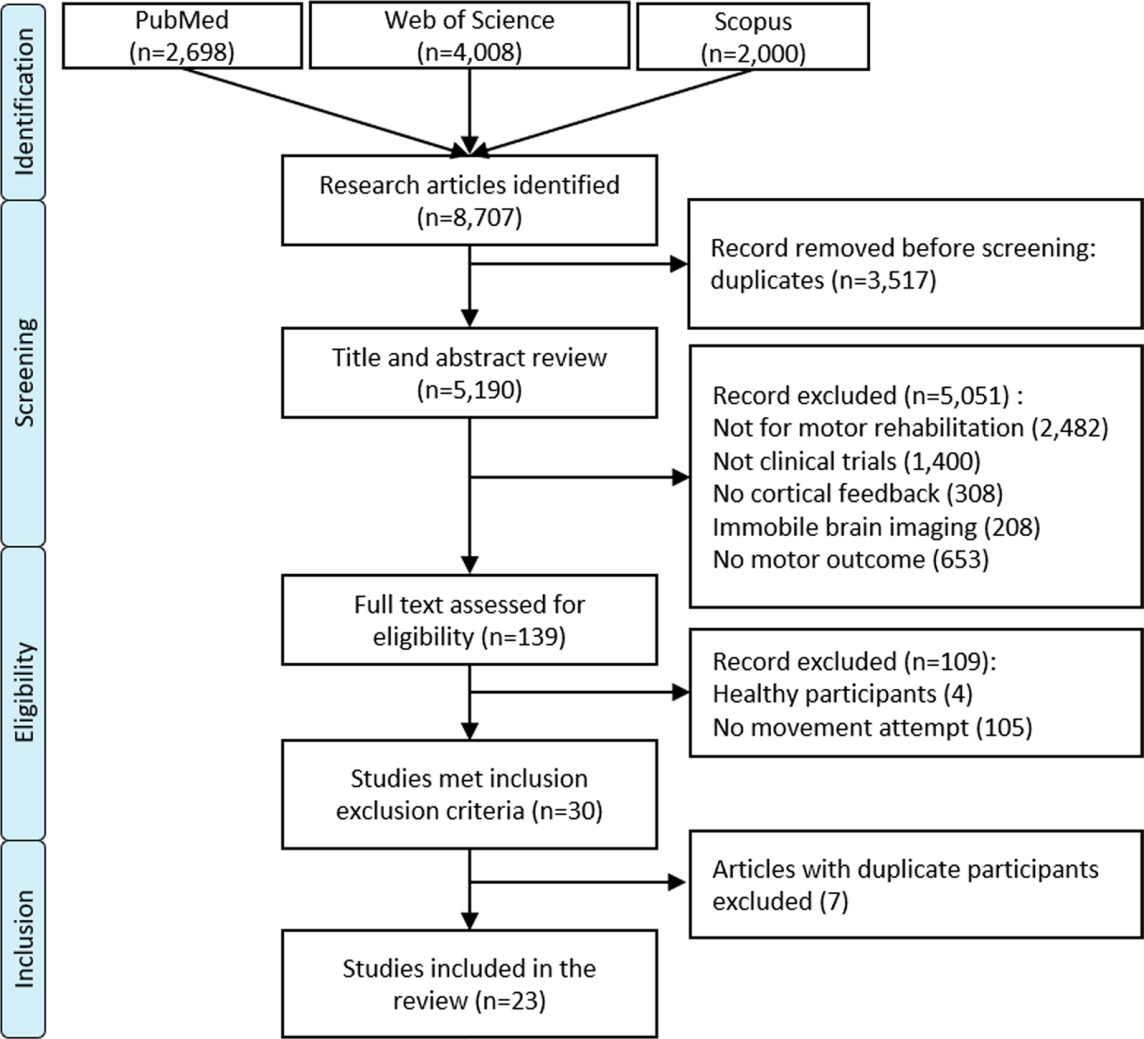
The PRISMA flow chart of eligibility assessment based on inclusion/exclusion criteria

Our search yielded 8707 citations across all databases (see Fig. 1 for details of the search result and entire screening process). After eliminating duplicates, 5190 unique articles remained. After title and abstract screening, 5051 articles were excluded. The full texts of the remaining 139 studies were reviewed with respect to inclusion-exclusion criteria. The criterion requiring the closest examination and generating the most discussion was whether participants were asked to attempt or imagine movement to elicit neurofeedback, regardless of whether or not they were able to perform the target movement on enrollment.

A total of 30 studies met all criteria and their data were extracted. During this process, it became apparent that some studies were from the same research group so were examined closely for any duplication of participants. Seven studies [41, 53–58] were conducted under the same clinical trial (registered at ClinicalTrails.gov, # NCT02098265) and used the same pool of subjects which varied by study; therefore, the most recent article with the most inclusive set of participants was retained, with the others excluded to avoid duplication. Similarly, outcome data from the original trial by Ramos-Murguialday et al. [4] were repeated to some extent in their long term follow-up study on 28 of the 30 original participants [5], the latter of which was therefore excluded. In contrast, although similar protocols were utilized by Cisotto et al. [59] and Silvoni et al. [60], both were included in the final list of studies because participants were unique to each study. The 2016 and 2019 RCTs by Mrachacz-Kersting et al. [26, 61] also used similar protocols but participants differed in the two studies, so both were included. The level of evidence, as defined by Sackett [62], varied across studies with none in Level I, six in Level II (small RCT) [4, 23, 26, 41, 63, 64], two in Level III [22, 61], eight in Level IV [59, 65–71], and seven in Level V (case study) [60, 72–77].

**Table 1:** Summary of participant and study design characteristics for all included studies

Study	Months post injury	Subject # (males)	Subject # NFT group	Task	Sessions	Control condition	Cointervention	Feedback type(s)	Functional outcomes	Sackett’s level
Bhagat (2020) [65]	9–106	10 (7)	10	Reaching	12	–	–	Robotic	FMA-UE, ARAT, GS, JTHF	IV
Biasiucci (2018) [22]	10	27 (16)	14	Wrist and finger extension	10	Sham FES	PT	FES	FMA-UE, MRC, MAS, ESS	III
Chen (2020) [23]	1–6	14 (12)	7	Wrist extension	12	MA with no FB	PT/OT/low frequency ES	Robotic	FMA-UE	II
Chowdhury (2018) [66]	6	4 (2)	4	Grasping	12–16	–	Robotic therapy	Robotic, Visual	ARAT, GS	IV
Chowdhury (2020) [67]	17–28	5 (2)	5	Grasping	12	–	Robotic therapy	Robotic, Visual	ARAT, GS	IV
Cisotto (2016) [59]	12	2 (2)	2	Reaching	10	–	–	Robotic, Visual	RTT	IV
Daly (2009) [72]	10	1 (0)	1	Finger extension	9	–	PT/OT/FES	FES, Visual	Volitional finger control	V
Ibáñez (2017) [68]	36–60	4 (4)	4	Reaching	8	–	–	FES	FMA-UE, SIS	V
Jang (2016) [63]	6	20 (10)	10	Shoulder ab/adduction	30	FES	OT	FES	MFT, MAS	II
Jovanovic (2020)[73]	72	1 (1)	1	Reaching and grasping	80	–	–	FES	FMA-UE, ARAT, FIM, TRI - HFT	V
Marquez-Chin (2016) [74]	72	1 (1)	1	Reaching	40	–	–	FES	FMA-UE, ARAT, FIM, TRI-HFT	V
McCrimmon (2015)[70]	6	9 (6)	9	Ankle dorsiflexion	12	–	–	FES	FMA-LE, 6MWD, Ankle ROM, Gait Speed	IV
Mrachacz- Kersting (2016) [61]	9–24	22 (19)	13	Ankle dorsiflexion	1	Sham FES	–	FES	10mWT, FMA-LE, AS, TAP frequency, NIH-SS	III
Mrachacz- Kersting (2019) [26]	4	24 (18)	12	Ankle dorsiflexion	12	70% motor threshold ES	-	FES	10 mWT, FMA-LE, modified RS, AS	II
Mukaino (2014) [75]	14	1 (1)	1	Finger extension (hand opening)	10	MA with No FB	OT	FES	FMA-UE, MAS	V
Norman (2018)[69]	6	8 (8)	8	Fingers extension (hand opening)	12	–	Robotic therapy	Visual	Box and Block Test	IV
Ono (2013) [76]	14	1 (1)	1	Fingers extension (hand opening)	15	FES	OT	FES	FMA-UE, MAS	V
Osuagwu (2016) [64]	3	12 (12)	7	Hand opening and closing	20	FES	PT/OT	FES, Visual	Wrist ROM, MMT	II
Ramos-Murguialday (2013) [4]	10	30 (18)	16	Hand opening and closing	20	Sham Robotic FB	PT	Robotic	combined FMA-UE, MAS, MAL, GAS	II
Remsik (2019)[41]	$\tilde{1}$–100	21 (9)	21	Grasping	9–15	Delayed Intervention	–	FES, Visual	ARAT, 9-HPT, NIH-SS, GS, SIS, Barthel Index	II
Silvoni (2013) [60]	$\tilde{1}08$	1 (0)	1	Reaching	6	–	–	Robotic, Visual	RTT	V
Takahashi (2012) [77]	30	1 (1)	1	Ankle dorsiflexion	1	FES	-	FES, Visual	Ankle ROM	V
Vourvopoulos (2019) [71]	6	4 (3)	4	Reaching (wrist and elbow extension)	8	–	–	Visual	FMA, SIS, MAS	IV

*FB* feedback; *FMA-UE* Fugl-Meyer Assessment Upper Extremity; *ARAT* Action Research Arm Test; *GS* Grip Strength; *MRC* Medical Research Council; *MAS* Modified Ashworth Scale; *FES* Functional Electrical Stimulation; *ES* Electrical Stimulation; *ESS* European Stroke Scale; *RTT* Reaction Time Test; *SIS* Stroke Impact Scale; *MFT* Muscle Function Test; *FIM* Functional Independence Measure; *TRI-HFT* Toronto Rehabilitation Institute Hand Function Test; *10mWT* 10-meter Walk Test; *6mWD* 6 Minute Walk Distance; *AS*= Ashworth scale; *mRS* modified Rankin Scale; *BBT* Box and Blocks Test; *cFMA* combined Fugl-Meyer Assessment; *MAL* motor activity log; *GAS* Goal Attainment Scale; *9-HPT* 9-Hole Peg Test; *NIH-SS* National Institutes of Health Stroke Scale; *MMT* Manual Muscle Test; *ROM*= Range of Motion; *OT* Occupational Therapy; *PT* Physical Therapy; *JBTH*=Jebsen Taylor Hand Function, *SCI* Spinal Cord Injury

### Participants

Table 1 provides a summary of details extracted from all included studies. A total of 223 individuals participated across studies; 153 of whom were in the BCI-NFT condition. All participants were adults, with 153 males and 70 females. Except for one study, on 12 participants with incomplete spinal cord injury, seven of whom were in the experimental group [64], the rest were on the stroke population. Nineteen studies only included participants more than 6 months post-stroke, and three studies, all RCTs, only focused on participants within the first 6 months of stroke; with 24 [26], 14 [23], and 20 participants [63]. Remsik et al., included four participants less than 6 months and 17 more than 6 months post-stroke [41]. Thus, in all there were 149 participants more than 6 months post stroke and 62 participants less than 6 months post stroke with 113 and 33 of those, respectively, participating in the BCI-NFT.

### Intervention

#### Motor task

The motor tasks targeted mostly involved the upper limb. Only four studies targeted the lower limbs [26, 61, 70, 77], all focused on ankle dorsiflexion. The upper limb motor tasks included shoulder ab/adduction (n = 1) [63], reaching (n = 5) [59, 60, 65, 68, 74], grasping (n = 5) [4, 41, 64, 66, 67], reaching and grasping (n = 2) [71, 73], hand and wrist extension (n = 2) [22, 23], and finger extension (n = 4) [69, 72, 75, 76].

#### Comparison conditions

Eight studies [4, 22, 23, 26, 41, 61, 63, 64] had a control or comparison condition, six of which were RCTs [4, 23, 26, 41, 63, 64]. The RCT by Remsik et al. used a delayed intervention period from 8 to 10 weeks as the control condition with nine participants then crossing over to the neurofeedback group [41]. In Chen et al., the control group attempted the motor task without BCI feedback [23]. In Jang et al. Functional electrical stimulation (FES) was delivered intermittently and was not driven by neural activity in controls with the target muscle group the same as for the experimental group [63]. In the Mrachacz-Kersting et al. 2019 RCT, both neurofeedback and control groups received electrical stimulation activated by the same cue-based BCI system, The intensity of stimulation in the control group, however, was much lower (%70 of motor threshold) than in the experimental group who received electrical stimulation at a functional level (at or above the motor threshold) [26]. In Ramos-Murguialday et al., sham robotic assistance, i.e., random movement of the robot not linked to brain activity, was the control [4]. For the cohort studies, [22, 61, 64] Mrachacz-Kersting et al. and Biasiucci et al. used “sham” FES, delivered randomly and not driven by neural activity, as the comparison [22, 61], whereas in Osuagwu et al. FES was delivered at set time intervals in the comparison condition [64]. Notably, Mrachacz-Kersting et al. changed the control condition from sham FES to low-intensity ES in their 2019 RCT. In two case reports by Takahashi et al. [77] and Ono et al. [76], subjects participated in both the neurofeedback (BCI driven FES) and comparison conditions (FES during motor attempt).

#### Dosage

The number of training sessions varied from 1 to 80 sessions, with six studies below 10 sessions [60, 61, 68, 71, 72, 77], 11 between 10 to 15 sessions [22, 23, 26, 41, 59, 65–67, 69, 70, 75], and six with more than 15 sessions [4, 63, 64, 73, 74, 76]. It is noteworthy that Takahashi et al. [77] and Mrachacz-Kersting et al. [61] demonstrated improvements after only one session of BCI-NFT.

#### Feedback type

Visual, robotic, or functional electrical stimulation were used during the motor training to augment voluntary effort and as additional sensory feedback. Fourteen studies used functional electrical stimulation (FES) alone (n = 10) [22, 26, 61, 63, 68, 70, 73–76], or combined with visual feedback (n = 4) [41, 64, 72, 77]. In a unique approach, Remsik et al. electrically stimulated the tongue along with visual and FES feedback to potentially enhance sensory input to cortical regions [41]. Robotic devices were used to provide proprioceptive feedback as well as motor assistance in seven studies, combined with visual feedback (n = 4) [59, 60, 66, 67] or alone in three studies [4, 23, 65]. Visual feedback was used alone in two studies [69, 71].

#### Neurofeedback paradigms

The summary of the experimental conditions and signal processing details can be found in Table 2. EEG was used in all 23 studies, likely due to its exceptional temporal resolution that enables this type of application. All studies had a calibration phase prior to training during which subjects attempted to execute the target task or to rest while EEG signals were recorded. These data were used to either identify the threshold between rest and motion in the selected features (n = 8) [4, 26, 61, 63, 71, 73, 74, 77], or to train a classifier (n = 15) using machine learning algorithms such as the linear classifier of BCI2000 software package (n = 5) [41, 59, 60, 69, 72], linear discriminant analysis (LDA; n = 5) [23, 64, 70, 75, 76], support vector machine (SVM; n = 3) [65–67], Gaussian classifier [22], or Logistic Regression Classifier [68]. During BCI-NFT, the trained classifiers or thresholds were used to detect when the brain activation indicated the motor attempt.

The BCI2000 software package, used in 5 studies, is a commercially available neurofeedback package which streamlines EEG processing by using the highest explained variance (r2) between the motor attempt and rest condition across all electrodes of interest and target brainwave frequency bins, i.e., a two-dimensional feature space. The electrodes (e.g., C3 and C4) and frequency bins (e.g., 10–12 Hz), that resulted in the highest r2 for each participant were selected and then fed into the BCI2000 linear classifier to generate the feedback command. In studies that used classifiers, a high number of electrodes, 12–47, were typically used to train the classifier. In studies not using BCI2000, a feature space was generated similarly with electrodes as one dimension and a range of frequency bins, often 2 or 3 Hz bins within the 8–30 Hz frequency band, as the other dimension for training the classifier, e.g., LDA or SVM. A subject-specific subset of this space would be used during the online classification for BCI detection of movement attempt.

In 17 studies, detection of the motor attempt triggered the feedback without controlling its intensity (go-no go) [4, 22, 23, 26, 41, 61, 63–65, 68, 70–76]. In two of those, Osuagwu et al. [64] and Remsik et al. [41], which had both proprioceptive and visual feedback, the visual feedback was modulated in a finite number of steps based on each BCI detection result. In three other studies [66, 67, 77], however, proprioceptive feedback intensity was increased in a finite number of steps, and in three studies, two proprioceptive [59, 60] and one visual feedback [69], feedback intensity was proportional to the brain signal intensity.

In Cisotto et al. [59] and Silvoni et al. [60] a robot provided assistive force proportional to ERD power of the selected subject-specific frequency band and, to promote concentration, visual feedback was provided based on the time it took participants to reach the target. In Norman et al. the brightness of a graphical object on the screen would change proportional to ERD power in the subject-specific frequency range [69]. In 2018 and 2020 studies by Chowdhury et al. [66, 67], detection of the hand opening and closing attempt, every 500 ms, triggered a robotic three-finger exoskeleton to open one step. Matching visualization of virtual hand motion was presented synchronously. Osuagwu et al. used a novel feedback strategy that enabled experimenters to adjust the difficulty level of the neurofeedback training [64] by adjusting the number of consecutive movement detections required for triggering FES.

Here almost all of the included studies except Mrachacz-Kersting et al. 2016 [61] and 2019 [26] and Bhagat et al. [65], which used MRCP (0.1–1 and 0.05–10 Hz, respectively) included frequencies in the Mu and/or Beta bands. The frequency ranges considered to be Mu and Beta bands differed slightly across studies and were in some cases truncated for the application. The majority of them (n = 12) included both Mu and Beta bands, referred to by some as the sensorimotor rhythms, or portions of each [22, 23, 59, 64, 66–68, 70–72, 75, 76]. Three studies only used the Mu band[4, 41, 73], four only used the Beta band [60, 69, 74, 77], and Jang et al. [63] used a combination of the Theta (4–7 Hz) and Beta bands.

Unlike classic neurofeedback studies as well as the many studies included here which used an operant conditioning paradigm, Mrachacz-Kersting et al. [26, 61] deployed an associative learning paradigm instead. They used the timing of the peak negativity, also referred to as contingent negative variation, in movement-related cortical potentials (MRCP), in the 0.05–10 Hz range of the EEG, to predict a dorsiflexion attempt and trigger FES assistance to the tibialis anterior muscle in a cue-based BCI-NFT system [78]. MRCP have several well-recognized and distinct features, including peak negativity, an electrical potential associated with movement planning around 500 ms before the motion [21]. Therefore, using this feature one can predict the movement, whereas in methods using ERD, detection usually occurs with the motor attempt, which may delay the triggering of a device. Predicting the movement as accurately as possible is a critical component of associative learning.

However, the protocol used in their studies [26, 61] also deviated from others in that the mean timing of the peak negativity was estimated in advance of the intervention and this fixed value was used during training, rather than real-time detection. An argument could be made that perhaps these studies should not have been included here because a feature of the brain activation was not utilized during the training sessions. However, we decided to retain them because the timing used in training was informed by individual brain data with the goal to improve the paradigm and these may offer a feasible and possibly more reliable solution than some alternatives for motor attempt paradigms, although the degree to which this improved detection was not assessed.

Other studies utilized other solutions in an effort to increase detection accuracy when MRCP are used. Ibanez et al. used two classifiers, Naïve Bayes and Match filter in the frequency and time domains, respectively, and combined results using a Logistic Regression Classifier to detect the reaching and grasping attempt and thereby trigger FES assistance to the anterior deltoids, triceps and wrist extensor muscles [68]. The frequency-domain classifier, Naïve Bayes, used ERD power in the 6–30 Hz frequency range to classify the motor attempt; the match filter used 0–1 Hz EEG signals , i.e., MRCP, to predict a motor attempt in a self-paced BCI.

**Table 2:** Narrative summary of procedures and related technical details utilized in the experimental condition of the included studies

Study	Experimental condition	Channels	Update rate	Pre-processing	Frequency range (Hz)	Real-time processing
Bhagat 2020 [65]	Participants instructed to first think about movement and then move. A center-out reaching task was performed using a robotic manipulandum with a graphical interface that presented targets requiring either elbow flexion (up arrow) or extension (down arrow). MRCP classifier detection corroborated by EMG triggered assistance; otherwise robot resisted motor attempts.	SMC 15 electrodes	–	–	MRCP (0.1–1)	SVM Classifier
Biasiucci 2018 [22]	Classifier built to differentiate active wrist and finger extension from rest. After a start cue was given, FES assistance was triggered. The threshold was optimized to avoid false positives so FES was never activated unless movement occurred.	SMC 16 electrodes	–	Laplacian	SMR (10–12) (18–24)	Gaussian Classifier
Chen 2020 [23]	Participants’ hands were inserted into a robotic force feedback device. A computer screen cued them when to move or to rest. BCI-detected activation indicating wrist extension attempt triggered the device to assist movement.	31 electrodes	–	CSP	Mu & Beta (8–30)	LDA Classifier
Chowdhury 2018 [66]	Participants first practiced opening and closing of thumb, index and middle fingers in assist-as-needed robotic device (physical practice). During the NF condition (mental practice), participants were instructed to as slowly as possible extend these fingers when cued from a computer screen showing a virtual hand grasping a remote. At 1.5 s after the cue and for the next 8500 ms intervals, each successful BCI detection triggered visual and robotic feedback of finger opening one step (up to 8 total steps).	SMC 12 electrodes	–	CSP	SMR (8–12 16–24)	SVM Classifier
Chowdhury 2020 [67]	Protocol similar to Chowdhury et al. 2018, see above, but with EMG electrodes placed on right and left forearm and collected synchronously with EEG to improve detection of finger opening.	10 EEG-EMG pairs SMC (12) + finger extension muscles (4)	–	CBPT	EEG: Mu (8–12) EMG: 30–50	SVM Classifier
Cisotto 2014 [59]	Participants performed a center-out reaching task using a robotic manipulandum with a graphical interface depicting targets in 4 directions. Targets to hit were shown in random order, and participants were cued to move to it within 500-740 ms. If successful, the target exploded; if too slow, it turned blue and if too fast, it turned red. ERD detection triggered robotic force assistance proportional to the ERD amplitude.	3 electrode-frequency pairs	8 ms	BCI2000	10–20	BCI2000 Linear Classifier
Daly 2009 [72]	Participant without index finger extension was instructed to attempt or alternatively imagine movement or relaxation. A red rectangle in the up position on the computer screen cued movement and one in the down position cued relaxation. Accurate detection of real or imagined movement triggered FES to assist finger extension. If ERD power threshold achieved during the task, rectangle turned green.	CP 3	–	BCI2000	5–30	BCI2000 Linear Classifier
Ibáñez 2017 [68]	Participants instructed to reach for a glass target in front of them at 75% of their maximum reach. BCI detection of movement intention triggered a multimodal FES activation reach sequence to assist them. Gyroscopes indicated whether movement occurred to evaluate accuracy of the BCI detection.	10 best electrode-frequency pair + a virtual channel average of C1,C2, and CZ	100 ms	Laplacian + ERD threshold identification	MRCP (0-1) & 6–35	(Naïve Bayes &Match filter) Logistic Regression Classifier
Jang 2016 [63]	Participants performed 4 shoulder movements demonstrated on a computer screen. A concentration index (CI), defined as EEG theta to beta power ratio was used as the detection threshold to trigger FES to two muscles that reduced shoulder subluxation during motor tasks.	FP1	-	CI Threshold identification	Beta (12–15) Theta (4–7)	–
Jovanovic 2020 [73]	Participants performed reaching, with or without hand opening when cued by a therapist. ERD detection of movement triggered FES to muscles that varied by participant and task. If detection did not occur, therapist could trigger FES.	C2	-	ERD/ERS measurement	Mu (8–12)	ERD Detection
Marquez-Chin 2016 [74]	Participant performed 5 reaching tasks (to mouth, opposite shoulder, knee on same side, in front and to the side), held the position, and returned to start. ERD detection of movement triggered a multichannel FES neuroprosthesis, if not, therapist could activate FES. Therapist also demonstrated and assisted movement as needed.	FCz	125 ms	ERD threshold identification	Beta (18–28)	ERD Detection
McCrimmon 2014 [70]	Participants attempted ankle dorsiflexion. BCI detection of movement intention triggered FES assistance. If FES not activated, they were to continue movement. If FES activated in error, they were told to not move.	1 subject-specific channel (CZ, C5 or CPz)	250 ms	CPCA	Mu & Beta (8–30)	LDA or AIDA
Mrachacz-Kersting 2016 [61] 2019 [26]	Participants instructed to dorsiflex as fast as possible once the cursor on the screen moved upward, hold for 2 s then relax. Timing of MRCP peak negativity during dorsiflexion attempt triggered FES assistance.	10 FP1, F3, F4, Fz, Pz, P3, P4, C3, C4, Cz	–	Laplacian	MRCP (0.5–10)	–
Mukaino 2014 [75]	Participants attempted to extend their fingers at maximum effort for 3s as demonstrated visually on computer screen. If ERD threshold reached for 1 s during the attempt FES assistance was triggered.	2 C3 and C4	30 ms	Laplacian	SMR (8–12 18–26)	LDA Classifier
Norman 2018 [69]	This study had 3 phases. Phase 1: participants attempted 1 of 4 cued commands to open only index, middle, both or neither fingers, with movement activating a finger extension robotic device. Phase 2: they practiced modulating SMR rhythms with success shown by increasing object brightness on a visual display. Phase 3: SMR modulation success as indicated by increasing object brightness, cued participants to move which in turn activated the robotic device.	1–3 electrode-frequency pairs	50 ms	BCI2000	Beta (12–24)	BCI2000 Linear Classifier
Ono 2013 [76]	Participants performed block trials of 5 hand openings for 3 s at maximal effort and 5 rests. If EEG classifier detected the hand opening attempt within 1s of cue to move, FES assistance was triggered.	2 C3 and C4	30 ms	–	Mu & Beta	LDA classifier
Osuagwu 2016 [64]	A buffer was established that set the number of consecutive successful BCI detections of either right- or left-hand movement required to trigger the FES. The buffer caused a needle on a gauge on a computer screen to point to 0 when the preset number was achieved and then triggered a multichannel FES to assist repetitive hand opening and closing . If the preset number for detection was not reached, no assistance occurred. Buffer size could be adjusted for difficulty.	Central SMC (3 bipolar electrodes, CP3- CF3, CPz–CFz, CP4–CF4)	–	–	Mu & Beta (7–30)	LDA Classifier
Ramos-Murguialday 2013 [4]	Participants performed either hand opening and closing, or moved the entire limb forward and backwards. If ERD was sustained below a threshold for 0.2 s during an attempt, a robotic exoskeleton was triggered to provide assistive force.	Ipsilesional motor cortex	-	ERD threshold identification	Mu (8–13)	ERD Detection
Remsik 2019 [41]	EEG classification of attempted grasping activated horizontal movement of a cursor on a screen, triggered FES assistance to facilitate finger opening or closing depending on participant choice and delivered contingent vibrotactile tongue stimulation.	C3–C4	-	BCI2000	Mu (8–12)	BCI2000 Linear Classifier
Silvoni 2013 [60]	Paradigm similar to Cisotto et al, 2016.	Right affected arm C3, CP1, P3, CP54 Left affected arm C4, CP2, P4, CP6	16 ms	BCI2000	Right affected arm 14–17 Hz Left affected arm 11–14 Hz	BCI2000 Linear Classifier
Takahashi 2012 [77]	Participant viewed a red square on a screen to cue ankle dorsiflexion. If ERD detection threshold reached, FES was triggered with stepwise increases or decreases every 500ms, depending on successful detection as shown by changing colors on 8 bars on the screen representing 8 possible steps.	Bipolar FCz-CPz	500 ms	ERD threshold identification	Beta 24–26	ERD Detection
Vourvopoulos 2019 [71]	Participants wore a head mounted VR display while instructed to attempt wrist and elbow extension. If EEG activation during attempt exceeded baseline, this triggered a virtual arm on a screen to move towards the target (visual FB).	2 C3, C4	500 ms	Laplacian	Mu and Beta 8–24	–

*MRCP* Movement-related cortical potential, *SVM* Support Vector Machine, *FES* Functional Electrical Stimulation, *SMR* Sensorimotor rhythm, *CSP* Common Spatial Patterns, *LDA* Linear Discriminant Analysis, *SMC* Sensorimotor cortex, *EMG* Electromyography, *EEG* Electroencephalography, *ERD* Event-related Desynchronization, *AIDA* Approximate Information Discriminant Analysis, *CBPT* Correlation of band-limited power time-courses

Bhagat et al. [65] and Chowdhury et al. [67] used electromyography (EMG) in combination with EEG to activate the feedback. Bhagat et al. trained an SVM classifier in the time domain using low frequency MRCP signals, 0.01 to 1 Hz, to detect motion; this detection, if corroborated by EMG activation, triggered the robotic assistive force [65]. Chowdhury et al. used four EMG channels for detection along with 12 EEG electrodes to create their feature space [67]. Only EEG-EMG channel pairs that showed a statistically significant correlation were selected as features for training an SVM classifier and thereby activating the feedback.

### Outcome analysis

Thirteen studies reported the mean FMA difference in the experimental group, pre to post intervention, ranging from 0.77 to 17.0 across studies, six of which showed significant improvements (see Table 3). The mean change across the 13 studies was 6.53 ± 4.46. Four studies additionally reported the FMA pre-post change score difference between the control and experimental groups [4, 22, 23, 26], two of which showed significantly greater improvements in the neurofeedback group [22, 26]. Mean change across the three studies was 4.06 ± 0.57. The mean Action Research Arm Test (ARAT) change score from pre to post intervention within the experimental group, reported in six studies [41, 65–67, 73, 74], ranged from 1.3 to 23.8 with the ARAT improvement significant in three [65–67]. Mean change across the six studies was 8.34 ± 9.00.

Several other motor outcomes were reported less frequently across studies. Grip strength (GS) was reported in five studies. Mean GS improvement, pre to post intervention, ranged from 3.87 kg [41] to 9.83 kg [67], both of which were statistically significant, p = 0.046 and p < 0.005, respectively. Mean GS post intervention increases were not significant in the other three studies [65, 66, 72]. Muscle spasticity was assessed in five studies using the Modified Ashworth Scale (MAS); however, no statistically significant changes were reported. Active dorsiflexion range of motion (ROM) improved in two studies [70, 77]: in McCrimmon et al., 5 of 9 participants showed an increase of $2.5^\circ$ or greater with a significant positive linear trend from pre to post intervention for the group as a whole (p < 0.01), and in Takahashi et al., the mean change of $8.7^\circ$ was also significant (p < 0.001) [77]. Mean active wrist extension ROM was improved by $16.8^\circ$ for the BCI-FES group versus $3.4^\circ$ for the FES group in the study by Osuagwu et al.; however, no statistical comparison was reported [64].

Mean Stroke Impact Scale (SIS) improvements of 5.4 [41], 10.5 [68] and 21.3 [71] were reported in three studies, none of which were significant. Despite no significant changes immediately post intervention, Remsik et al. [41] showed a significant SIS improvement of 6.2 (p = 0.05) at follow-up. A small, statistically significant improvement in gait speed of 0.08 m/s (p = 0.007) was reported only in the neurofeedback group by Mrachacz-Kersting et al. (2016), as measured by the 10 Meter Walk Test (10mWT) after only 20 min (30 pairs of MA and associative feedback) of neurofeedback training [61]. McCrimmon et al. in contrast showed no significant improvement in walking speed in the neurofeedback group [70] after 12 training sessions; the linear trend from pre to post intervention, however, was significantly positive. Mrachacz-Kersting et al. (2019) found significant 10mWT improvements in the control and neurofeedback groups (both p < 0.008) with no statistical between group difference [26]. Further, they reported that five participants in the experimental group and three in the control group who could not walk pre-intervention were able to walk after 12 training sessions [26]. Significant 10mWT improvements were also found in the control group with no between group difference. All other motor outcomes reported in each study are listed in Table 1. The trends for these outcomes were mostly positive or unchanged except for reaction time, which worsened significantly after training [59, 60].

**Table 3:** Summary of the Fugl-Meyer Assessment (FMA) results for the Upper Extremity (UE) and the Lower Extremity (LE) and the Action Research Arm Test (ARAT) results reported in the individual studies for the Neurofeedback Training (NFT) and Control (C) groups

Study	FMA-UE (NFT)	FMA-UE (NFT vs C)	FMA-LE (NFT)	FMA-LE (NFT vs C)	ARAT (total, NFT)	ARAT (sub-scores)
Bhagat 2020 [65]	3.92*	–	–	–	5.35*	–
Biasiucci 2018 [22]	6.7*	4.6*	–	–	–	–
Chen 2020 [23]	8.42*	3.71	–	–	–	–
Chowdhury 2018 [66]	–	–	–	–	5.66*	–
Chowdhury 2020 [67]	–	–	–	–	23.75*	–
Ibáñez 2017 [68]	11.5	–	–	–	–	–
Jovanovic 2020 [73]	17*	–	–	–	14*	Grasp: 3; Grip: 8; Pinch: 1; Gross Move: 4
Marquez- Chin 2016 [74]	6	–	–	–	0	–
McCrimmon 2014 [70]	–	–	2.44	–	–	–
Mrachacz- Kersting 2016 [61]	–	–	0.77*	–	–	–
Mrachacz- Kersting 2019 [26]	–	–	8.5*	4.5*	–	–
Mukaino 2014 [75]	8	–	–	–	–	–
Ono 2013 [76]	7	–	–	–	–	–
Ramos- Murguialday 2013 [4]	3.40	3.45	–	–	–	–
Remsik 2019 [41]	–	–	–	–	1.3*	Grasp: 0.7; Grip: 0.1; Pinch: 0.4; Gross Move: 0
Vourvopoulos 2019 [71]	1.25	–	–	–	–	–
Mean	7.32	3.92	3.90	4.5	8.34	–
Standard Deviation	4.46	0.60	4.07	–	9.00	–

*FMA-UE* Fugl-Meyer Assessment Upper Extremity, *FMA-LE* Fugl-Meyer Assessment Lower Extremity, *ARAT* Action Research Arm Test, *NFT* Neurofeedback Training Group, *C* Control Group

Significant values (p < 0.05) indicated by Asterisk

### Statistical analyses

Independent t-tests were conducted to assess the effect of feedback type (robotic: n = 3, and FES: n = 9) and co-interventions (yes or no) on FMA differences within experimental groups in the 13 studies which reported these data. Despite higher mean FMA values for FES compared to robotic feedback (7.6 ± 4.8 vs 5.3 ± 2.8; p = 0.47), outcomes were not significantly better. Conversely, the ARAT mean difference was higher for robotic (n = 3) vs FES feedback (n = 3) (11.59 ± 10.53 vs 5.10 ± 7.34), but was also not significant. The inclusion of co-interventions showed no consistent or statistically significant effect on FMA and ARAT scores (for FMA: none=6.4 ± 5.6 [n = 9], yes = 6.7 ± 2.04 [n=4]; p = 0.92, for ARAT: none=5.16 ± 6.32 [n = 4], yes = 14.7 ± 12.8 [n=2]; p = 0.47). Mean FMA change also did not differ significantly by the level of evidence as assessed with a general linear model (II = 6.8 ±2.4 (n = 4), III = 0.77 (n = 1), IV = 4.8 ± 4.6 (n = 4), V = 9.5 ± 5.1 (n = 4); p = 0.27).

The effect of differences between classic versus associative learning paradigms on FMA within the experimental group was also assessed using an independent t-test. The two studies that used associative learning [26, 61] had a slightly lower mean FMA score when compared with studies that used operant conditioning (n=8) [4, 22, 23, 65, 68, 70, 71, 75, 76] of 4.6 vs 6.9, respectively. The difference, however, was not significant (p = 0.54).

Pearson correlation between FMA improvement after BCI-NFT and the number of training sessions showed a moderate positive relationship (r = 0.67, p = 0.01). Despite a similar correlation value between ARAT score and the number of sessions; this was not statistically significant (r = 0.70, p = 0.20).

## Discussion

Similar to generally positive trends from other reviews assessing clinical effectiveness of BCI-NFT paradigms, all studies identified here reported largely positive, albeit not always statistically significant, motor outcomes. Our meta-analyses demonstrated that the FMA mean change in the experimental group exceeded the minimal clinically important difference (MCID) of 5.5 points as did 8 of the 13 studies reporting this; however, none of values from the 4 studies which subtracted the control group mean reached the MCID. Only one of six studies reporting the ARAT showed a value that exceeded the MCID (i.e. >17 points). We identified a dose response with a greater number of sessions directly and moderately related to greater effectiveness with 12 sessions the maximal number of sessions in studies with FMA results. Given this, it is possible that more prolonged training would produce even larger effects. While outcomes did not vary significantly based on level of evidence, designs that include control groups should be strongly encouraged as should blinding of outcomes, so that there can be far greater confidence in the results that are reported. Another key consideration in rehabilitation is the persistence of effects beyond the training period. Long term effects were assessed in only four studies [22, 41, 65, 70]; overall the improvements persisted at follow up, which ranged from 1 [70] to 36 weeks [22]. The improvement in ARAT lasted at least 4 weeks in Remsik et al. [41]. Bhagat et al, demonstrated that FMA-Upper Extremity (UE) and ARAT scores remained significantly higher than baseline 2 weeks and 2 months post intervention [65]. More notable, however, was the follow-up period in Biasiucci et al., where significant improvement in FMA-UE and Medical Research Council were maintained 9 months post intervention [22]. In addition to the included studies, Ramos-Murguialday et al. [5] conducted a follow-up study to their 2013 study [4] (included in this review) using the same participants and demonstrated significant FMA, Motor Activity Log and Goal Attainment Scale improvements which lasted more than 6 months post intervention.

There were no clear study design features other than session number that altered the magnitude of positive effects. We were particularly interested in whether the type of feedback provided influenced the motor outcome. In animal models, the role of muscle spindle feedback is crucial for locomotor recovery and spinal circuit reorganization, and is presumed to also be important in humans [79]. Both FES and robotic movement assistance during BCI-NFT paradigms provide proprioceptive input because they elicit or augment muscle stretch which thereby activates muscle spindles, Golgi tendon organs and cutaneous receptors [5, 24, 26, 33, 80]. However, in unloaded conditions, (e.g. weight support provided by a robotic device), proprioceptive signaling relies almost exclusively on muscle spindles [79]. Ono et al. [81] showed the superiority of proprioceptive feedback, provided by a hand robot, to visual feedback in a cohort study of 12 stroke patients. Although robotic feedback can provide afferent proprioceptive feedback and has been used extensively [4, 24, 33] FES depolarizes more motor and sensory axons, thus should provide greater proprioceptive feedback [82]. Therefore, we expected to find that FES feedback was superior; however, in our sample, there was no difference in effectiveness between FES and robotic feedback. This is in contrast with the Bai et al. systematic review, which demonstrated that FES had a significantly larger effect on functional recovery than visual and robotic feedback [42]. Some of the studies here also incorporated visual feedback of detection success which can further upregulate the reward system in the brain and thereby enhance motor learning [83]. The presence of cointerventions also did not significantly augment effectiveness, suggesting that these alone did not account for the positive outcomes. The more recent associative learning paradigms failed to demonstrate better outcomes. No other training features were prevalent enough for statistical comparisons.

Most studies on BCI-NFT for motor rehabilitation are focused on the stroke population, despite their potential benefit for other nondegenerative neurological disorders such as CP, which is the most common motor disorder in the pediatric population. It has been shown that individuals with CP can self-regulate their brain activity to control BCI systems to activate assistive devices [84, 85]. Two BCI-NFT studies on the CP population, excluded here because they utilized motor imagery, aimed to improve hand function by self-regulation, i.e., reduction of mu band activity. Bobrov et al., utilizing a hand exoskeleton for feedback, trained 14 children with CP [36]. Significant gains in hand function were found for the FMA, ARAT and Jebsen-Taylor Test, after 7–10 weeks of training. This protocol was previously used by their group for training patients post-stroke in multiple studies [86–88]. The second study in CP [89] showed a decrease (improvement) in a serial reaction time task with the non-dominant hand, after only three sessions of BCI-NFT (8 min each) using visual feedback. Motor attempt paradigms that directly link movement associated brain signals to external devices and do not require them to actively try to modulate brain activity or to consistently imagine a specific movement make BCI-training far more feasible and accessible to a broader range of patients, even very young children.

One observation from our review is that the terminology used across studies to describe the intervention is not consistent. The term “neurofeedback” has been used extensively in clinical applications, including motor rehabilitation, particularly those that involve operant conditioning [34, 48, 56, 59, 61, 63, 66, 71, 89–92]; however, this term alone was not sufficient for a comprehensive literature search. We found it necessary to also search for BCI and BMI terms, which greatly increased the yield of our search strategy but also resulted in a very large numbers of excluded studies. BCI systems have a broad range of applicability including for activating robotic, prosthetic or communication devices and enhancing cognitive functioning in disorders such as attention deficit hyperactivity disorder (ADHD) [16] or post-stroke [46] by training them to self-regulate their cortical activity. Some authors referred to motor training applications as rehabilitative or restorative BCIs, to contrast these with assistive BCI for those who lack movement capabilities. One very recent study included here [21] did not use either term (neurofeedback or BCI) but instead used the term “brain state-dependent stimulation” to describe their system for retraining motor function that did not include the BCI component in this case to illustrate that it is the precisely timed afferent volley that is the essential component for changes in cortical excitability. Here they used a pre-determined timing of peripheral nerve stimulation delivery with respect to the cue, instead of real-time detection, calculated from previously collected MRCP data. While we used the term “BCI-neurofeedback” here to encompass the two main terms used in the preponderance of studies on rehabilitation applications, motor rehabilitation paradigms have diverged from classic operant conditioning neurofeedback paradigms and as they continue to evolve, may warrant new more relevant descriptors.

Motor attempt paradigms demonstrated several consistent features across studies. Although fNIRS is commonly used for mobile brain imaging, all studies here used EEG. Even within the 139 full texts we reviewed, only three used fNIRS for their BCI [90, 91, 93]. This is similar to a recent review by Mane et al. [46] on BCI application for stroke rehabilitation, in which 47 of 50 studies used EEG alone with one using EEG plus MEG [94], one used MEG [95] and one used fNIRS [90]. EEG benefits from a far higher temporal resolution than fNIRS [48, 96],and less expensive and more accessible than MEG, and therefore, it has been used almost exclusively in BCI-NFT applications.

The precise temporal association between the afferent sensory feedback and the motor command was deemed to be the reason for significant functional improvements in several studies [22, 61]. The effect of stimulation timing was evaluated by Mrachacz-Kersting et al. using healthy participants [97] and they found that when the timing of stimulation delivery was either before or after the motor planning phase of the MRCP, which typically occurs within 500 ms of movement onset [21], no plasticity was induced. Using precise temporal association, this group demonstrated significant functional improvements after a single session consisting of 30–50, motor attempt-FES pairings for about 20 min [61] in those more than 6 months post stroke which is remarkable since the median range for BCI-NFT protocols here was 10–15 sessions. For FMA-Lower Extremity (LE), significant changes were even larger and reached the MCID after 12 sessions of training [26], in their later (2019) study. The synchronous activation of the motor cortex and peripheral effectors may induce plasticity using the principle of Hebbian associativity; and thereby, strengthen the connectivity of the corticospinal tract with the sensory and motor cortices. This was evaluated in both of their studies, by measuring the motor evoked potential (MEP) using TMS; corticospinal excitability was significantly higher only in the experimental group 30 seconds post-intervention [26, 61]. Although associative learning paradigms were not shown here to be more effective, the numbers of studies are limited; therefore, the jury is still out and more comparative data are needed. Biasiucci et al. [22] also considered the time contingency between motor decoding and FES as the main reason for their impressive clinical improvement, which lasted at least 30 weeks post intervention. Using EEG data as an outcome measure they verified the hypothesized enhanced functional connectivity in the affected sensorimotor cortex post-intervention.

### Recommendations for the field

The small number of studies with the same functional outcomes included in this review limited the ability to identify specific protocols or features with superior efficacy or effectiveness. Many paradigms aimed to produce reliable (minimal false positives or negatives) but varied in the timing of activation of an external assistive device with movement onset, ideally recommended to occur within a 300 ms window [98]. Therefore, it seems reasonable that paradigms that use EEG activity prior to movement to predict movement intention rather than real-time detection of movement onset would be preferable, if not essential, considering the time delays related to EEG processing and communication between system software and hardware. MRCP contain signals that precede movement; however, the time between these and movement onset or device activation can fluctuate within and across individuals, and perhaps are even more variable for those with brain injuries. To account for this, machine leaning algorithms, such as the Gaussian classifier utilized by Biasiucci et al. [22], and Naïve Bayes deployed by Ibanez et al. [68] that can predict or classify the motor attempt online, might be a more precise alternative than relying on the consistency of the MRCP signals such as peak negativity timing with respect to motor onset. These could reduce the calibration time and thereby optimize therapy time, and account more effectively for individual variability, This could perhaps be improved even further using transfer learning algorithms to train a predictive model once and then transfer this across participants, thus eliminating the calibration phase [99]. Proprioceptive feedback was used almost exclusively for these motor rehabilitation applications, rather than visual feedback alone, which is logical since these also serve assistive as well as sensory-enhancing roles. More data on the short- and longer-term efficacy of FES compared to robotic feedback are still needed. Modulation and progression or weaning of feedback over time are important future considerations to maximize motor learning and neuroplasticity. While we did not compare effectiveness of motor imagery to motor attempt, the latter is more intuitive and clearly more feasible across a broader range of patients.

### Limitations

Some limitations are that this scoping review did not, by design, include all studies on the use of BCI-NFT, but it does provide a comprehensive review on the current state of the science on motor attempt paradigms for neurorehabilitation. Another possible limitation is that we did not restrict the studies by the level of evidence; however, significant mean differences in outcomes across levels were not found. The limited use of consistent outcomes across studies also restricts the number of studies included in any meta-analysis, reinforcing the need for greater efforts in rehabilitation research to enable the accumulation of larger datasets with common data definitions and outcomes. Still, given the limitations, several studies demonstrated clinically significant functional changes after short durations of training, far shorter than typically needed for producing the same magnitude of effects with motor training alone.

### Conclusion

In conclusion, the specific focus on enhancing neuroplasticity within a task-specific paradigm with BCI-NFT provides a solid neurophysiological mechanism for potential behavioral changes that we believe are only beginning to be realized. Future efforts should be directed towards deploying these in younger patient populations with greater neuroplastic potential and designing these systems for broad clinical implementation and for larger efficacy trials that compare these to other forms of neuromodulation or other evidence-based motor training approaches at equivalent doses.

## Data Availability

Not applicable.

## Abbreviations

10mWT: 10 meter walk test
ADHD: Attention deficit hyperactivity disorder
ARAT: Action research arm test
BCI: Brain computer interface
CP: Cerebral Palsy
EEG: Electroencephalogram
EMG: Electromyogram
ERD: Event related desynchronization
FES: Functional electrical stimulation
FMA: Fugl-Meyer assessment
GS: Grip strength
GLM: General linear model
LDA: Linear discriminant analysis
LE: Lower extremity
MCID: Minimal clinically important difference
MEG: Magnetoencephalography
MEP: Motor evoked potential
MI: Motor imagery
MRCP: Movement-related cortical potentials
MRI: Magnetic resonance imaging
RCT: Randomized controlled trials
ROM: Range of motion
SIS: Stroke impact scale
SVM: Support vector machine
tDCS: Transcranial direct current stimulation
TMS: Transcranial magnetic stimulation
UE: Upper extremity

## References

[1] Tariq M, Trivailo PM, Simic M. Eeg-based bci control schemes for lower-limb assistive-robots. Front Human Neurosci. 2018;312. 10.3389/fnhum.2018.00312 PMC6088276 30127730

[2] Baniqued PDE, Stanyer EC, Awais M, Alazmani A, Jackson AE, Mon-Williams MA, Mushtaq F, Holt RJ (2021). Brain–computer interface robotics for hand rehabilitation after stroke: a systematic review. J Neuroeng Rehabil.

[3] Abiri R, Borhani S, Sellers EW, Jiang Y, Zhao X (2019). A comprehensive review of eeg-based brain–computer interface paradigms. J Neural Eng.

[4] Ramos-Murguialday A, Broetz D, Rea M, Läer L, Yilmaz O, Brasil FL, Liberati G, Curado MR, Garcia-Cossio E, Vyziotis A, Cho W, Agostini M, Soares E, Soekadar S, Caria A, Cohen LG, Birbaumer N (2013). Brain–machine interface in chronic stroke rehabilitation: a controlled study. Ann Neurol.

[5] Ramos-Murguialday A, Curado MR, Broetz D, Yilmaz O, Brasil FL, Liberati G, Garcia-Cossio E, Cho W, Caria A, Cohen LG, Birbaumer N (2019). Brain–machine interface in chronic stroke: randomized trial long-term follow-up. Neurorehabil Neural Repair.

[6] Sonuga-Barke E, Brandeis D, Holtmann M, Cortese S (2014). Computer-based cognitive training for adhd: a review of current evidence. Child Adolesc Psychiatr Clin.

[7] Evans JR, Budzynski TH, Budzynski HK, Abarbanel A (2009). Introduction to quantitative EEG and neurofeedback: advanced theory and applications.

[8] Hurt E, Arnold LE, Lofthouse N (2014). Quantitative eeg neurofeedback for the treatment of pediatric attention-deficit/hyperactivity disorder, autism spectrum disorders, learning disorders, and epilepsy. Child Adolesc Psychiatr Clin.

[9] McCarthy-Jones S (2012). Taking back the brain: could neurofeedback training be effective for relieving distressing auditory verbal hallucinations in patients with schizophrenia?. Schizophr Bull.

[10] Othmer S. Progress in neurofeedback for the autism spectrum. In: 38th Annual Meeting of the Association for Applied Psychophysiology Biofeedback. Monterey, Canada, pp. 15–18.

[11] Horrell T, El-Baz A, Baruth J, Tasman A, Sokhadze G, Stewart C, Sokhadze E (2010). Neurofeedback effects on evoked and induced eeg gamma band reactivity to drug-related cues in cocaine addiction. J Neurother.

[12] Hammer BU, Colbert AP, Brown KA, Ilioi EC (2011). Neurofeedback for insomnia: a pilot study of z-score smr and individualized protocols. Appl Psychophysiol Biofeedback.

[13] Walker JE (2010). Using qeeg-guided neurofeedback for epilepsy versus standardized protocols: enhanced effectiveness?. Appl Psychophysiol Biofeedback.

[14] Ibric VL, Dragomirescu LG. Neurofeedback in pain management. Introduction to quantitative EEG and neurofeedback: Advanced theory and applications 2nd edn., 2009;417–451.

[15] Marzbani H, Marateb HR, Mansourian M (2016). Neurofeedback: a comprehensive review on system design, methodology and clinical applications. Basic Clin Neurosci.

[16] Sitaram R, Ros T, Stoeckel L, Haller S, Scharnowski F, Lewis-Peacock J, Weiskopf N, Blefari ML, Rana M, Oblak E (2017). Closed-loop brain training: the science of neurofeedback. Nat Rev Neurosci.

[17] Motolese F, Capone F, Di Lazzaro V (2022). New tools for shaping plasticity to enhance recovery after stroke. Handb Clin Neurol.

[18] O’Leary GH, Jenkins DD, Coker-Bolt P, George MS, Kautz S, Bikson M, Gillick BT, Badran BW (2021). From adults to pediatrics: a review noninvasive brain stimulation (nibs) to facilitate recovery from brain injury. Prog Brain Res.

[19] Thompson AK, Wolpaw JR (2015). Targeted neuroplasticity for rehabilitation. Prog Brain Res.

[20] Wolpaw JR (2018). The negotiated equilibrium model of spinal cord function. J Physiol.

[21] Mrachacz-Kersting N, Ibáñez J, Farina D. Towards a mechanistic approach for the development of non-invasive brain–computer interfaces for motor rehabilitation. J Physiol. 2021. 10.1113/jp281314. 10.1113/JP281314 33728656

[22] Biasiucci A, Leeb R, Iturrate I, Perdikis S, Al-Khodairy A, Corbet T, Schnider A, Schmidlin T, Zhang H, Bassolino M, Viceic D, Vuadens P, Guggisberg AG, Millán JDR (2018). Brain-actuated functional electrical stimulation elicits lasting arm motor recovery after stroke. Nat Commun.

[23] Chen S, Cao L, Shu X, Wang H, Ding L, Wang SH, Jia J (2020). Longitudinal electroencephalography analysis in subacute stroke patients during intervention of brain–computer interface with exoskeleton feedback. Front Neurosci.

[24] Frolov AA, Mokienko O, Lyukmanov R, Biryukova E, Kotov S, Turbina L, Nadareyshvily G, Bushkova Y (2017). Post-stroke rehabilitation training with a motor-imagery-based brain-computer interface (bci)-controlled hand exoskeleton: A randomized controlled multicenter trial. Front Neurosci.

[25] Miao YY, Chen SG, Zhang XR, Jin J, Xu R, Daly I, Jia J, Wang XY, Cichocki A, Jung TP. Bci-based rehabilitation on the stroke in sequela stage. Neural Plasticity. 2020;2020. 10.1155/2020/8882764. 10.1155/2020/8882764 PMC7752268 33414824

[26] Mrachacz-Kersting N, Stevenson AJ, Jørgensen HR, Severinsen KE, Aliakbaryhosseinabadi S, Jiang N, Farina D (2019). Brain state-dependent stimulation boosts functional recovery following stroke. Ann Neurol.

[27] Brain functional networks study of subacute stroke patients with upper limb dysfunction after comprehensive rehabilitation including bci training. Front Neurol . 2019;10:1419. 10.3389/fneur.2019.01419. 10.3389/fneur.2019.01419 PMC7000923 32082238

[28] Khan MA, Das R, Iversen HK, Puthusserypady S (2020). Review on motor imagery based bci systems for upper limb post-stroke neurorehabilitation: from designing to application. Comput Biol Med.

[29] Lazarou I, Nikolopoulos S, Petrantonakis PC, Kompatsiaris I, Tsolaki M (2018). Eeg-based brain–computer interfaces for communication and rehabilitation of people with motor impairment: a novel approach of the 21 (st) century. Front Hum Neurosci.

[30] Wen D, Fan Y, Hsu SH, Xu J, Zhou Y, Tao J, Lan X, Li F (2021). Combining brain–computer interface and virtual reality for rehabilitation in neurological diseases: a narrative review. Ann Phys Rehabil Med.

[31] Millán JD, Rupp R, Müller-Putz GR, Murray-Smith R, Giugliemma C, Tangermann M, Vidaurre C, Cincotti F, Kübler A, Leeb R, Neuper C, Müller KR, Mattia D. Combining brain–computer interfaces and assistive technologies: state-of-the-art and challenges. Front Neurosci. 2010;4. 10.3389/fnins.2010.00161. 10.3389/fnins.2010.00161 PMC2944670 20877434

[32] Bockbrader MA, Francisco G, Lee R, Olson J, Solinsky R, Boninger ML (2018). Brain computer interfaces in rehabilitation medicine. PMR.

[33] Ang KK, Chua KSG, Phua KS, Wang C, Chin ZY, Kuah CWK, Low W, Guan C (2015). A randomized controlled trial of eeg-based motor imagery brain–computer interface robotic rehabilitation for stroke. Clin EEG Neurosci.

[34] Shindo K, Kawashima K, Ushiba J, Ota N, Ito M, Ota T, Kimura A, Liu M (2011). Effects of neurofeedback training with an electroencephalogram-based brain–computer interface for hand paralysis in patients with chronic stroke: a preliminary case series study. J Rehabil Med.

[35] Várkuti B, Guan C, Pan Y, Phua KS, Ang KK, Kuah CW, Chua K, Ang BT, Birbaumer N, Sitaram R (2013). Resting state changes in functional connectivity correlate with movement recovery for bci and robot-assisted upper-extremity training after stroke. Neurorehabil Neural Repair.

[36] Bobrov P, Biryukova E, Polyaev B, Lajsheva O, Usachjova E, Sokolova A, Mihailova D, Dement’Eva K, Fedotova I. Rehabilitation of patients with cerebral palsy using hand exoskeleton controlled by brain–computer interface. Bull Russian State Med Univ. 2020;(4).

[37] Blankertz B, Losch F, Krauledat M, Dornhege G, Curio G, Müller KR (2008). The berlin brain–computer interface: accurate performance from first-session in bci-naïve subjects. IEEE Trans Biomed Eng.

[38] Nijholt A, Tan D, Pfurtscheller G, Brunner C, Millán JdR, Allison B, Graimann B, Popescu F, Blankertz B, Müller K-R (2008). Brain–computer interfacing for intelligent systems. IEEE Intell Syst.

[39] Zhang X, Guo Y, Gao B, Long J (2020). Alpha frequency intervention by electrical stimulation to improve performance in mu-based bci. IEEE Trans Neural Syst Rehabil Eng.

[40] Blokland Y, Vlek R, Karaman B, Özin F, Thijssen D, Eijsvogels T, Colier W, Floor-Westerdijk M, Bruhn J, Farquhar J (2012). Detection of event-related desynchronization during attempted and imagined movements in tetraplegics for brain switch control. Annu Int Conf IEEE Eng Med Biol Soc.

[41] Remsik AB, Williams JL, Gjini K, Dodd K, Thoma J, Jacobson T, Walczak M, McMillan M, Rajan S, Young BM, Nigogosyan Z, Advani H, Mohanty R, Tellapragada N, Allen J, Mazrooyisebdani M, Walton LM, van Kan PLE, Kang TJ, Sattin JA, Nair VA, Edwards DF, Williams JC, Prabhakaran V (2019). Ipsilesional mu rhythm desynchronization and changes in motor behavior following post stroke bci intervention for motor rehabilitation. Front Neurosci.

[42] Bai Z, Fong KNK, Zhang JJ, Chan J, Ting KH (2020). Immediate and long-term effects of bci-based rehabilitation of the upper extremity after stroke: a systematic review and meta-analysis. J Neuroeng Rehabil.

[43] Muralidharan A, Chae J, Taylor DM (2011). Extracting attempted hand movements from eegs in people with complete hand paralysis following stroke. Front Neurosci.

[44] Muralidharan A, Chae J, Taylor DM. Early detection of hand movements from electroencephalograms for stroke therapy applications. J Neural Eng. 2011;8(4). 10.1088/1741-2560/8/4/046003. 10.1088/1741-2560/8/4/046003 PMC3148608 21623009

[45] Jeunet C, Glize B, McGonigal A, Batail JM, Micoulaud-Franchi JA (2019). Using eeg-based brain computer interface and neurofeedback targeting sensorimotor rhythms to improve motor skills: theoretical background, applications and prospects. Neurophysiol Clin.

[46] Mane R, Chouhan T, Guan C (2020). Bci for stroke rehabilitation: motor and beyond. J Neural Eng.

[47] Kohl SH, Mehler DMA, Lührs M, Thibault RT, Konrad K, Sorger B (2020). The potential of functional near-infrared spectroscopy-based neurofeedback—a systematic review and recommendations for best practice. Front Neurosci.

[48] Pichiorri F, Mattia D (2020). Brain–computer interfaces in neurologic rehabilitation practice. Handb Clin Neurol.

[49] Carvalho R, Dias N, Cerqueira JJ (2019). Brain–machine interface of upper limb recovery in stroke patients rehabilitation: a systematic review. Physiother Res Int.

[50] Cervera MA, Soekadar SR, Ushiba J, Millán JdR, Liu M, Birbaumer N, Garipelli G (2018). Brain–computer interfaces for post-stroke motor rehabilitation: a meta-analysis. Annal Clin Transl Neurol.

[51] Tricco AC, Lillie E, Zarin W, O’Brien KK, Colquhoun H, Levac D, Moher D, Peters MD, Horsley T, Weeks L (2018). Prisma extension for scoping reviews (prisma-scr): checklist and explanation. Ann Intern Med.

[52] Peters MD, Godfrey C, McInerney P, Baldini Soares C, Khalil H, Parker D, Munn Z. Chapter 11: scoping reviews. Joanna Briggs Institute Reviewer’s Manual. The Joanna Briggs Institute; 2017.

[53] Young BM, Nigogosyan Z, Walton LM, Remsik A, Song J, Nair VA, Tyler ME, Edwards DF, Caldera K, Sattin JA, Williams JC, Prabhakaran V. Dose-response relationships using brain–computer interface technology impact stroke rehabilitation. Front Human Neurosci. 2015;9. 10.3389/fnhum.2015.00361. 10.3389/fnhum.2015.00361 PMC4477141 26157378

[54] Young BM, Stamm JM, Song J, Remsik AB, Nair VA, Tyler ME, Edwards DF, Caldera K, Sattin JA, Williams JC, Prabhakaran V (2016). Brain–computer interface training after stroke affects patterns of brain-behavior relationships in corticospinal motor fibers. Front Hum Neurosci.

[55] ...Remsik AB, Dodd K, Leroy JW, Thoma J, Jacobson T, Allen JD, Advani H, Mohanty R, McMillan M, Rajan S, Walczak M, Young BM, Nigogosyan Z, Rivera CA, Mazrooyisebdani M, Tellapragada N, Walton LM, Gjini K, Van Kan PLE, Kang TJ, Sattin JA, Nair VA, Edwards DF, Williams JC, Prabhakaran V. Behavioral outcomes following braincomputer interface intervention for upper extremity rehabilitation in stroke: a randomized controlled trial. Front Neurosci. 2018;12. 10.3389/fnins.2018.00752. 10.3389/fnins.2018.00752 PMC6235950 30467461

[56] Young BM, Nigogosyan Z, Nair VA, Walton LM, Song J, Tyler ME, Edwards DF, Caldera K, Sattin JA, Williams JC, Prabhakaran V (2014). Case report: post-stroke interventional bci rehabilitation in an individual with preexisting sensorineural disability. Front Neuroeng.

[57] Young BM, Nigogosyan Z, Remsik A, Walton LM, Song J, Nair VA, Grogan SW, Tyler ME, Edwards DF, Caldera K (2014). Changes in functional connectivity correlate with behavioral gains in stroke patients after therapy using a brain–computer interface device. Front Neuroeng.

[58] Young BM, Nigogosyan Z, Walton LM, Song J, Nair VA, Grogan SW, Tyler ME, Edwards DF, Caldera K, Sattin JA (2014). Changes in functional brain organization and behavioral correlations after rehabilitative therapy using a brain–computer interface. Front Neuroeng.

[59] Cisotto G, Pupolin S, Cavinato M, Piccione F (2014). An eeg-based bci platform to improve arm reaching ability of chronic stroke patients by means of an operant learning training with a contingent force feedback. Int J E-Health Med Commun.

[60] Silvoni S, Cavinato M, Volpato C, Cisotto G, Genna C, Agostini M, Turolla A, Ramos-Murguialday A, Piccione F (2013). Kinematic and neurophysiological consequences of an assisted-force-feedback brain–machine interface training: a case study. Front Neurol.

[61] Mrachacz-Kersting N, Jiang N, Thomas Stevenson AJ, Niazi IK, Kostic V, Pavlovic A, Radovanovic S, Djuric-Jovicic M, Agosta F, Dremstrup K, Farina D (2016). Efficient neuroplasticity induction in chronic stroke patients by an associative brain–computer interface. J Neurophysiol.

[62] Sackett DL (1989). Rules of evidence and clinical recommendations on the use of antithrombotic agents. Chest.

[63] Jang YY, Kim TH, Lee BH (2016). Effects of brain–computer interface-controlled functional electrical stimulation training on shoulder subluxation for patients with stroke: A randomized controlled trial. Occup Ther Int.

[64] Osuagwu BC, Wallace L, Fraser M, Vuckovic A (2016). Rehabilitation of hand in subacute tetraplegic patients based on brain computer interface and functional electrical stimulation: a randomised pilot study. J Neural Eng.

[65] Bhagat NA, Yozbatiran N, Sullivan JL, Paranjape R, Losey C, Hernandez Z, Keser Z, Grossman R, Francisco GE, O’Malley MK, Contreras-Vidal JL (2020). Neural activity modulations and motor recovery following brain-exoskeleton interface mediated stroke rehabilitation. Neuroimage Clin.

[66] Chowdhury A, Meena YK, Raza H, Bhushan B, Uttam AK, Pandey N, Hashmi AA, Bajpai A, Dutta A, Prasad G (2018). Active physical practice followed by mental practice using bci-driven hand exoskeleton: a pilot trial for clinical effectiveness and usability. IEEE J Biomed Health Inform.

[67] Chowdhury A, Dutta A, Prasad G (2020). Corticomuscular co-activation based hybrid brain–computer interface for motor recovery monitoring. Ieee Access.

[68] Ibáñez J, Monge-Pereira E, Molina-Rueda F, Serrano JI, Del Castillo MD, Cuesta-Gómez A, Carratalá-Tejada M, Cano-de-la-Cuerda R, Alguacil-Diego IM, Miangolarra-Page JC, Pons JL (2017). Low latency estimation of motor intentions to assist reaching movements along multiple sessions in chronic stroke patients: a feasibility study. Front Neurosci.

[69] Norman SL, McFarland DJ, Miner A, Cramer SC, Wolbrecht ET, Wolpaw JR, Reinkensmeyer DJ (2018). Controlling pre-movement sensorimotor rhythm can improve finger extension after stroke. J Neural Eng.

[70] McCrimmon CM, King CE, Wang PT, Cramer SC, Nenadic Z, Do AH (2014). Brain-controlled functional electrical stimulation for lower-limb motor recovery in stroke survivors. Annu Int Conf IEEE Eng Med Biol Soc.

[71] Vourvopoulos A, Pardo OM, Lefebvre S, Neureither M, Saldana D, Jahng E, Liew SL (2019). Effects of a brain–computer interface with virtual reality (vr) neurofeedback: a pilot study in chronic stroke patients. Front Hum Neurosci.

[72] Daly JJ, Cheng R, Rogers J, Litinas K, Hrovat K, Dohring M (2009). Feasibility of a new application of noninvasive brain computer interface (bci): a case study of training for recovery of volitional motor control after stroke. J Neurol Phys Ther.

[73] Jovanovic LI, Kapadia N, Lo L, Zivanovic V, Popovic MR, Marquez-Chin C (2020). Restoration of upper limb function after chronic severe hemiplegia: a case report on the feasibility of a brain–computer interface-triggered functional electrical stimulation therapy. Am J Phys Med Rehabil.

[74] Marquez-Chin C, Marquis A, Popovic MR (2016). Eeg-triggered functional electrical stimulation therapy for restoring upper limb function in chronic stroke with severe hemiplegia. Case Rep Neurol Med.

[75] Mukaino M, Ono T, Shindo K, Fujiwara T, Ota T, Kimura A, Liu M, Ushiba J (2014). Efficacy of brain–computer interface-driven neuromuscular electrical stimulation for chronic paresis after stroke. J Rehabil Med.

[76] Ono T, Mukaino M, Ushiba J (2013). Functional recovery in upper limb function in stroke survivors by using brain–computer interface a single case a-b-a-b design. Annu Int Conf IEEE Eng Med Biol Soc.

[77] Takahashi M, Takeda K, Otaka Y, Osu R, Hanakawa T, Gouko M, Ito K (2012). Event related desynchronization-modulated functional electrical stimulation system for stroke rehabilitation: a feasibility study. J Neuroeng Rehabil.

[78] Mrachacz-Kersting N, Jiang N, Dremstrup K, Farina D. A novel brain-computer interface for chronic stroke patients. In: Christoph Guger ECL, Brendan Allison (eds) Brain–computer interface research: a state-of-the-art summary 2. Biosystems and Biorobotics, vol. 6, pp. 51–61. Springer, Berlin, Heidelberg 2014. 10.1007/978-3-642-54707-2_6.

[79] Takeoka A, Vollenweider I, Courtine G, Arber S (2014). Muscle spindle feedback directs locomotor recovery and circuit reorganization after spinal cord injury. Cell.

[80] Sebastián-Romagosa M, Cho W, Ortner R, Murovec N, Von Oertzen T, Kamada K, Allison BZ, Guger C. Brain computer interface treatment for motor rehabilitation of upper extremity of stroke patients-a feasibility study. Front Neurosci. 2020;14. 10.3389/fnins.2020.591435. 10.3389/fnins.2020.591435 PMC7640937 33192277

[81] Ono T, Shindo K, Kawashima K, Ota N, Ito M, Ota T, Mukaino M, Fujiwara T, Kimura A, Liu M, Ushiba J (2014). Brain–computer interface with somatosensory feedback improves functional recovery from severe hemiplegia due to chronic stroke. Front Neuroeng.

[82] Bergquist A, Clair J, Lagerquist O, Mang C, Okuma Y, Collins D (2011). Neuromuscular electrical stimulation: implications of the electrically evoked sensory volley. Eur J Appl Physiol.

[83] Bradley CL, Damiano DL (2019). Effects of dopamine on motor recovery and training in adults and children with nonprogressive neurological injuries: a systematic review. Neurorehabil Neural Repair.

[84] Cruz A, Pires G, Lopes A, Carona C, Nunes UJ (2021). A self-paced bci with a collaborative controller for highly reliable wheelchair driving: Experimental tests with physically disabled individuals. IEEE Trans Human-Mach Syst.

[85] Daly I, Billinger M, Laparra-Hernández J, Aloise F, García ML, Faller J, Scherer R, Müller-Putz G (2013). On the control of brain–computer interfaces by users with cerebral palsy. Clin Neurophysiol.

[86] Bobrov P, Frolov AA, Husek D. In: Kudelka M, Pokorny J, Snasel V, Abraham A (eds) Brain Computer Interface Enhancement by Independent Component Analysis. Advances in Intelligent Systems and Computing, vol. 179, 2013;51–60. 10.1007/978-3-642-31603-6_5. url:$$<$$Go to ISI$$>$$://WOS:000312116400005.

[87] Frolov AA, Bobrov PD, Biryukova EV, Silchenko AV, Kondur AA, Dzhalagoniya IZ, Massion J (2018). Electrical, hemodynamic, and motor activity in bci post-stroke rehabilitation: clinical case study. Front Neurol.

[88] Kotov SV, Turbina LG, Bobrov PD, Frolov AA, Pavlova OG, Kurganskaia ME, Biriukova EV (2014). rehabilitation of post stroke patients using a bioengineering system “brain–computer interface + exoskeleton”. Zh Nevrol Psikhiatr Im S S Korsakova.

[89] alves-Pinto A, Turova V, Blumenstein T, Hantuschke C, Lampe R (2017). Implicit learning of a finger motor sequence by patients with cerebral palsy after neurofeedback. Appl Psychophysiol Biofeedback.

[90] Mihara M, Hattori N, Hatakenaka M, Yagura H, Kawano T, Hino T, Miyai I (2013). Near-infrared spectroscopy-mediated neurofeedback enhances efficacy of motor imagery-based training in poststroke victims: a pilot study. Stroke.

[91] Fujimoto H, Mihara M, Hattori N, Hatakenaka M, Yagura H, Kawano T, Miyai I, Mochizuki H (2017). Neurofeedback-induced facilitation of the supplementary motor area affects postural stability. Neurophotonics.

[92] Rayegani S, Raeissadat S, Sedighipour L, Mohammad Rezazadeh I, Bahrami M, Eliaspour D, Khosrawi S (2014). Effect of neurofeedback and electromyographic-biofeedback therapy on improving hand function in stroke patients. Top Stroke Rehabil.

[93] Ding Q, Lin T, Wu M, Yang W, Li W, Jing Y, Ren X, Gong Y, Xu G, Lan Y (2021). Influence of itbs on the acute neuroplastic change after bci training. Front Cell Neurosci.

[94] Caria A, Weber C, Brötz D, Ramos A, Ticini LF, Gharabaghi A, Braun C, Birbaumer N (2011). Chronic stroke recovery after combined bci training and physiotherapy: a case report. Psychophysiology.

[95] Buch E, Weber C, Cohen LG, Braun C, Dimyan MA, Ard T, Mellinger J, Caria A, Soekadar S, Fourkas A, Birbaumer N (2008). Think to move: a neuromagnetic brain–computer interface (bci) system for chronic stroke. Stroke.

[96] Gwin JT, Gramann K, Makeig S, Ferris DP (2011). Electrocortical activity is coupled to gait cycle phase during treadmill walking. Neuroimage.

[97] Mrachacz-Kersting N, Kristensen SR, Niazi IK, Farina D (2012). Precise temporal association between cortical potentials evoked by motor imagination and afference induces cortical plasticity. J Physiol.

[98] Grosse-Wentrup M, Mattia D, Oweiss K (2011). Using brain–computer interfaces to induce neural plasticity and restore function. J Neural Eng.

[99] Raza H, Chowdhury A, Bhattacharyya S. Deep learning based prediction of eeg motor imagery of stroke patients’ for neuro-rehabilitation application. In: 2020 International Joint Conference on Neural Networks (IJCNN), pp. 1–8. IEEE.

